# What is interdisciplinarity, and how can we apply it to research and practice?

**DOI:** 10.1093/eurpub/ckac129.229

**Published:** 2022-10-25

**Authors:** M Freye

**Affiliations:** 1 IGMR, University of Bremen, Bremen, Germany; 2 Leibniz ScienceCampus Digital Public Health, Bremen, Germany

## Abstract

The concept of interdisciplinarity is an integral part of contemporary academia. Successful academic careers or innovative study programs: Interdisciplinarity is often claimed, expected, or required. Public health scientists also claim to facilitate health-related interdisciplinary research under the umbrella of public health and to apply findings from interdisciplinary research in practice. These aspects already point to one of many problems in using the term interdisciplinarity: When using the term, it is not always evident whether it is a merely descriptive term or whether normative expectations are also associated with it. We consider that successful interdisciplinary research is enabled by a shared understanding of what is meant by interdisciplinarity in concrete research projects. This urgency is re-emphasised by the participation of new disciplines in digital public health. Through this presentation, we would like to give participants a brief theoretical overview of the concept of interdisciplinarity and related concepts such as transdisciplinarity and multidisciplinarity. In this way, all workshop participants will have a shared understanding that will serve as a basis for further work in groups during the workshop. After the terminological distinctions, we will turn to the practical challenges of interdisciplinary research, which arise beyond the clarification of terms. We will outline these challenges in fundamental terms and then provide some best practice examples on how interdisciplinarity in scientific competence can be promoted among individual scientists and in research groups. In the best case, the workshop participants will draw inspiration for the subsequent group work from the best practice examples to generate new motivation for interdisciplinary work in this way. A concise summary will conclude this compact-informative presentation. Merle Freye will host the tables on law and ethics during the world coffee.

